# A One-year Hospital System Review of Plasma Next-Generation Sequencing in a Mixed Population

**DOI:** 10.1017/ash.2024.220

**Published:** 2024-09-16

**Authors:** Ty Drake, Jessica Babic, Audrey Wanger, Violeta Chavez

**Affiliations:** Memorial Hermann Hospital; Memorial Hermann-Texas Medical Center; UT Health McGovern Medical School

## Abstract

Objective: To describe whether detecting plasma microbial cell-free DNA by next-generation sequencing (NGS) provided additional information compared to routine cultures or led to change in antimicrobial management. Design and setting: This is a retrospective cohort study evaluating NGS tests performed on patients who were admitted to an 11-acute care hospital health system in the greater Houston area between May 2022 and May 2023. Repeat tests on the same patient encounter were included if >7 days from previous test. Routine microbiology data was compared if test was collected within 7 days before or after NGS testing. **Results:** During the study period there were 135 unique patient encounters identified with an NGS order. Of which, 74.1% were ≥18 years of age and 46.7% were immunocompromised. A total of 143 NGS tests were ordered, with 4 not being run due to quality control issues. Out of 139 NGS tests completed, 76 (54.7%) were positive for at least one organism. When compared to routine testing, NGS alone was positive in 29 (20.9%) instances, routine testing alone was positive in 17 (12.2%) instances, both were positive in 44 (31.7%) instances, and both were negative in 49 (35.3%) instances. In the 44 instances that both NGS and routine testing were positive, 15 (34.1%) were concordant for all organisms. In total, NGS identified 92 more organisms (69 bacterial, 8 fungal, and 15 viral) compared to routine testing and routine testing identified 42 more organisms (28 bacterial, 6 fungal, 11 viral, and 1 parasite) compared to NGS. Fifty-six changes to antibiotic therapy were made within 48 hours of the NGS test resulting, with 16 of these changes being directly attributed to NGS test. Nine of these changes being escalations and seven being de-escalations. **Conclusion:** NGS may aid in determining further testing, earlier detection of pathogens, and detection of pathogens outside of routine testing resulting in direct changes to antimicrobials. However, results need to be interpreted with caution. NGS can miss pertinent pathogens and is difficult to interpret when commensal organisms are detected, both of which can lead to unnecessary testing or treatment. There is an absence of a clear algorithm for the use of NGS testing and the test comes with a high price and unclear utility. Further studies are needed to determine which patients may most benefit from NGS testing.

